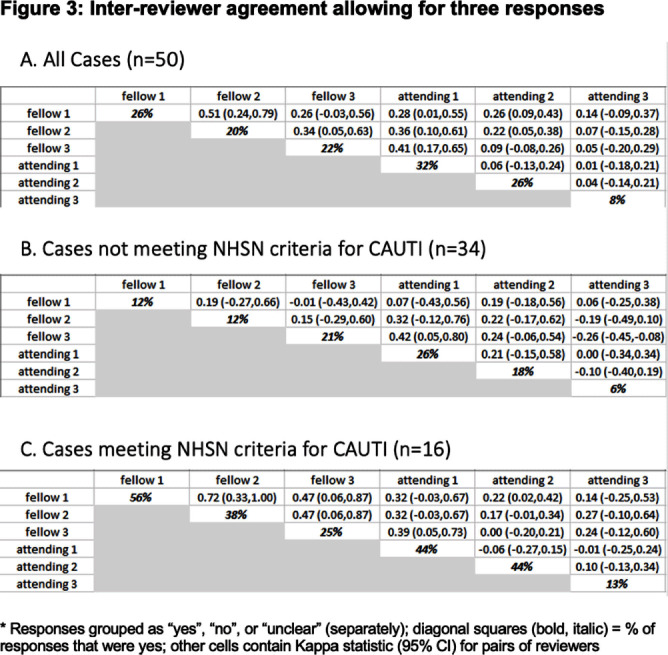# Inter-rater agreement of CAUTI (catheter-associated urinary tract infections) diagnosis among Infectious disease physicians

**DOI:** 10.1017/ash.2024.197

**Published:** 2024-09-16

**Authors:** Divya Kondapi, James Wang, Francisco Rincon, Lizy Paniagua, Mohammed Raja, Bhavarth Shukla, Hayley Gershengorn

**Affiliations:** University of South Florida; University of Tennessee Medical Center at Knoxville; MercyOne Des Moines Medical Center; Mount Sinai Medical Center; Univeristy of Miami; University of Miami Health System; University of Miami Miller School of Medicine

## Abstract

**Background:** CAUTIs constitute forty percent of nosocomial infections, yet their direct link with mortality remains debated. In 2009, NHSN estimated the economic burden of CAUTIs in the U.S. to be over $340 million. Limited data exist on inter-physician concordance in diagnosing CAUTIs, especially in patients with abnormal genitourinary (GU) anatomy. Our study assessed inter-provider variability in diagnosing CAUTI in 50 such patients, including those meeting NHSN(National healthcare safety network) criteria. **Methods:** We included a random set of 50 adults (18+) with abnormal GU anatomy admitted to the University of Miami hospitals from January 2018 to November 2021 who had a urinary foley catheter and at least one positive urine culture during their hospitalization. Three Infectious disease fellows and three board-certified Infectious disease physicians independently reviewed each patient’s chart, classifying them as having or not having a CAUTI. Inter-physician reliability was assessed using kappa statistics. **Results:** Our findings highlight substantial variation in clinician-determined CAUTI incidence among the 50 patients with abnormal GU anatomy, ranging from 8% to 32% (Figures 2,3). Inter-rater agreement on CAUTI diagnosis was generally poor (Kappa Hollenbeak CS, et al. The attributable cost of catheter-associated urinary tract infections in the United States: A systematic review. Am J Infect Control. 2018 Jul;46(7):751-757. Trautner BW, et al. Development and validation of an algorithm to recalibrate mental models and reduce diagnostic errors associated with catheter-associated bacteriuria. BMC Med Inform Decis Mak. 2013 Apr 15;13:48. Gafary M, et al. Catheter Associated Urinary Tract Infections (CAUTI) in Bladder Cancer Patients Post Cystectomy With a Neobladder, Open Forum Infectious Diseases, Volume 2, Issue suppl_1, December 2015, 293.

**Figure:**
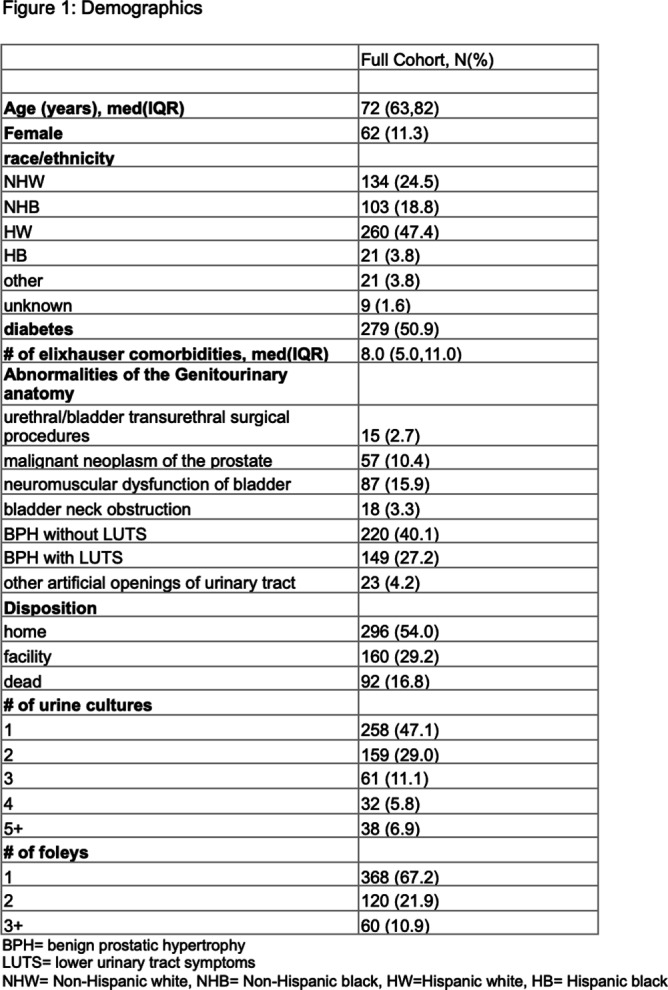

**Figure:**
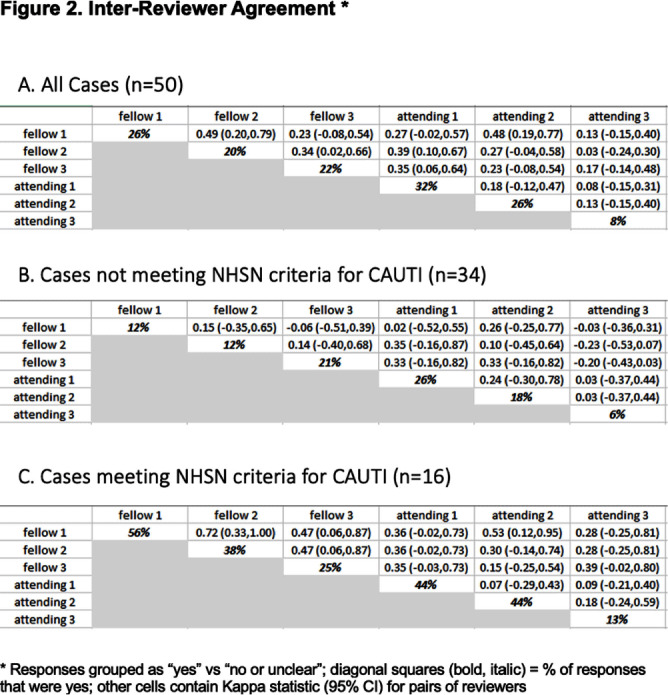

**Figure:**